# Prospective case control study of iron deficiency and the risk of febrile seizures in children in South Korea

**DOI:** 10.1186/s12887-019-1675-4

**Published:** 2019-09-04

**Authors:** Han Na Jang, Hoi Soo Yoon, Eun Hye Lee

**Affiliations:** Department of Pediatrics, School of Medicine, Kyung Hee University, 23, Kyung Hee Dae-ro, Dongdaemun-gu, Seoul, 130-872 South Korea

**Keywords:** Anemia, Children, Febrile seizures, Ferritin, Iron deficiency

## Abstract

**Background:**

Febrile seizures are the most common type of seizure in the first 5 years of life, and many factors that increase seizure risk have been identified. This study was performed to examine the association between iron status and febrile seizures in children in South Korea.

**Methods:**

A prospective unmatched case control study was performed in 63 cases of febrile seizures and 65 controls with febrile illness but no seizures.

**Results:**

Serum iron, plasma ferritin, and transferrin saturation were significantly lower in children with febrile seizures compared to the controls. Iron deficiency, defined as ferritin < 30 ng/mL, was more prevalent in the febrile seizure group (49.2%) than in the control group (16.9%). Serum iron < 22 ng/dL (odds ratio 3.42, 95% confidence interval [CI] 1.31–8.9, *P* = 0.012) and ferritin < 30 ng/mL (odds ratio 6.18, 95% CI 2.32–16.42, *P* < 0.001) were associated with increased risk of developing febrile seizures in multivariate logistic regression analysis.

**Conclusion:**

These observations suggest that iron deficiency prior to development of anemia may increase risk of febrile seizures.

## Introduction

Febrile seizures are defined as seizures accompanied by fever without central nervous systemic infection or metabolic disorder. It is the most common type of seizure in the first 5 years of life, which affect 2–5% of all children [1]. Children with simple febrile seizures usually have a good prognosis, with no evidence of increased rates of mortality, hemiplegia, or cognitive deficits [2]. Previous studies identified various risk factors for febrile seizures, including developmental delay, discharge from a neonatal unit after 28 days, daycare attendance, viral infections, family history of febrile seizures, certain vaccinations, and nutritional deficiencies, including iron and zinc [3–8]. The prevalence rate of febrile seizures differ between regions. The recently reported 5 year prevalence of febrile seizures in South Korea was 6.92%, which is slightly higher than mean prevalence of 2–5% of all children in worldwide [9]. Although it is a benign condition, their patients and family may have very frightening experience and high levels of anxiety. In Korean culture, many parents seek oriental medicine for febrile seizures, where they may receive unidentified herbal medicine or acupuncture to their young children [10]. It is therefore important to determine the preventable risk factors and give adequate information for their parents to prevent unnecessary interventions to children with febrile seizures.

Iron is an important nutrient that acts as a cofactor for several enzymes in the body, as well as playing roles in the production and function of neurotransmitters, hormones, and DNA (deoxyribonucleic acid) duplication. Iron is also essential for enzymes involved in neurochemical reactions, such as myelin formation, metabolism of some neurotransmitters, and brain energy metabolism [11]. Iron deficiency anemia is associated with behavioral abnormalities and impaired cognitive function. It has potential for irreversible brain damage if it occurs during the most active period of brain development in young children [12].

Reports regarding the association between febrile seizures and iron status have been inconsistent; some studies indicated that iron deficiency with or without anemia was more prevalent in children with febrile seizures [13–18], whereas others found no association between iron deficiency and febrile seizures [19–21]. Although many studies have dealt this issue, iron deficiency anemia, however, is just as important because it is a widespread nutritional problem and can be prevented by screening and clinical concerns. Nevertheless, almost all of these previous studies were conducted in the Middle East, particularly in Iran and Pakistan, with only a few such studies performed in other parts of the world. Because the iron status and prevalence of iron deficiency anemia is highly related to socioeconomic state, malnutrition, weaning practices, which is highly dependent on cultural and geographic differences [22], the association of febrile seizures and iron deficiency anemia may vary region to region.

Here, we compared the iron status of children with febrile seizures and controls to investigate the association between iron status and febrile seizures in children in South Korea.

## Methods

This prospective case control study was performed between August 2015 and July 2017. The study population consisted of 128 patients aged 6 to 60 months admitted to the Department of Pediatrics of Kyung Hee University Hospital (Seoul, South Korea). Sixty-three children with febrile seizures and 65 controls with febrile illness only were included in the study. The parents of all patients provided written informed consent for inclusion in the study, which was approved by the Medical Sciences Ethics Committee of Kyung Hee University Hospital.

The febrile seizure group (*n* = 63) included patients with seizure accompanied by fever ≥38 °C without central nervous system infection or metabolic disorders. The control group (*n* = 65) was selected randomly from among children admitted for febrile illnesses, such as gastroenteritis, otitis media, or respiratory tract infections, without seizure around the same time with the cases. Patients with chronic cardiovascular, renal, rheumatological or malignant diseases, and hemoglobinopathies, or other blood disorders were excluded from the study as they were more likely to have anemia. Patients with central nervous system diseases such as developmental delay, motor disabilities, and mental or cognitive defects were also excluded as they could have nutritional deficiency that may affect the results of the study. All of the febrile seizure patients and controls received appropriate diets for their ages without feeding problems. The febrile seizure and control groups were comparable in age, gender distribution, and clinical characteristics of febrile illness. Routine hematologic investigation were performed at the emergency department or 1st day of admission. The laboratory results regarding blood indices and iron status were analyzed using complete blood count (CBC), serum iron, plasma ferritin, total iron binding capacity (TIBC), and transferrin saturation, which were compared between the two groups. Patients were diagnosed with complex febrile seizures if they had experienced prolonged (> 15 min in duration), focal, or repetitive (more than one seizure within 24 h) seizures [23]. The laboratory variables were compared between patients with complex and simple febrile seizures.

Anemia was defined as a hemoglobin (Hb) level of 2 standard deviations below the normal values for age, i.e., Hb < 10.5 g/dL for ages 6–24 months and < 11.5 g/dL for ages 2–5 years. Iron deficiency was defined as serum iron < 22 μg/dL, plasma ferritin < 30 ng/mL, or transferrin saturation < 16% [24, 25].

Children with a history of afebrile seizures, any antiepileptic drug medication, central nervous system infection, neurological deficit, or developmental delay were excluded from the study.

A pilot study on 60 patients (24 cases and 34 controls) was performed for sample size estimation. Using G*power 3.1, based on α = 0.05 and study power (1–β) = 0.8, mean ferritin level 38.4 ± 20.5 ng/mL (cases) and 60.9 ± 53.1 ng/mL (control), the sample size of each group was estimated 52.

**Fig. 1:**
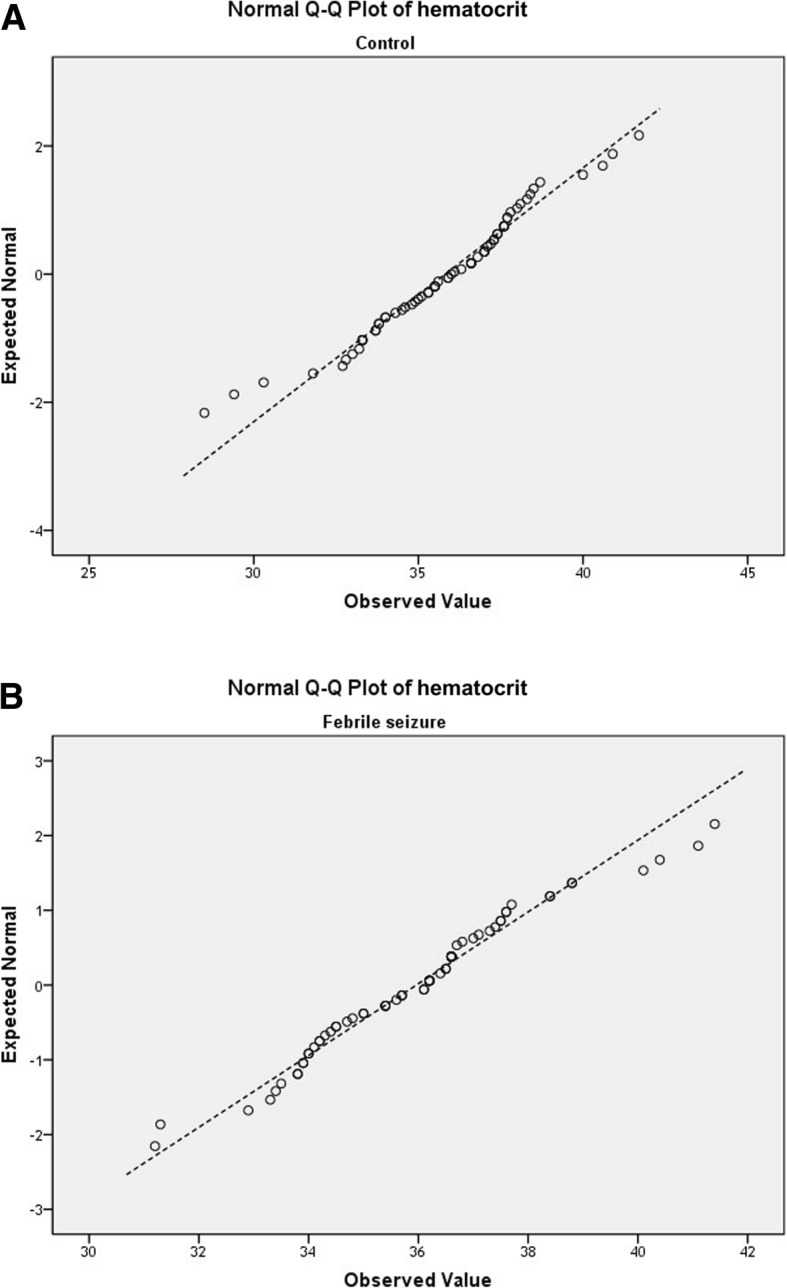
Q-Q plots of hematocrit level in control group (**a**) and febrile seizure group (**b**) shows normal distribution

**Fig. 2:**
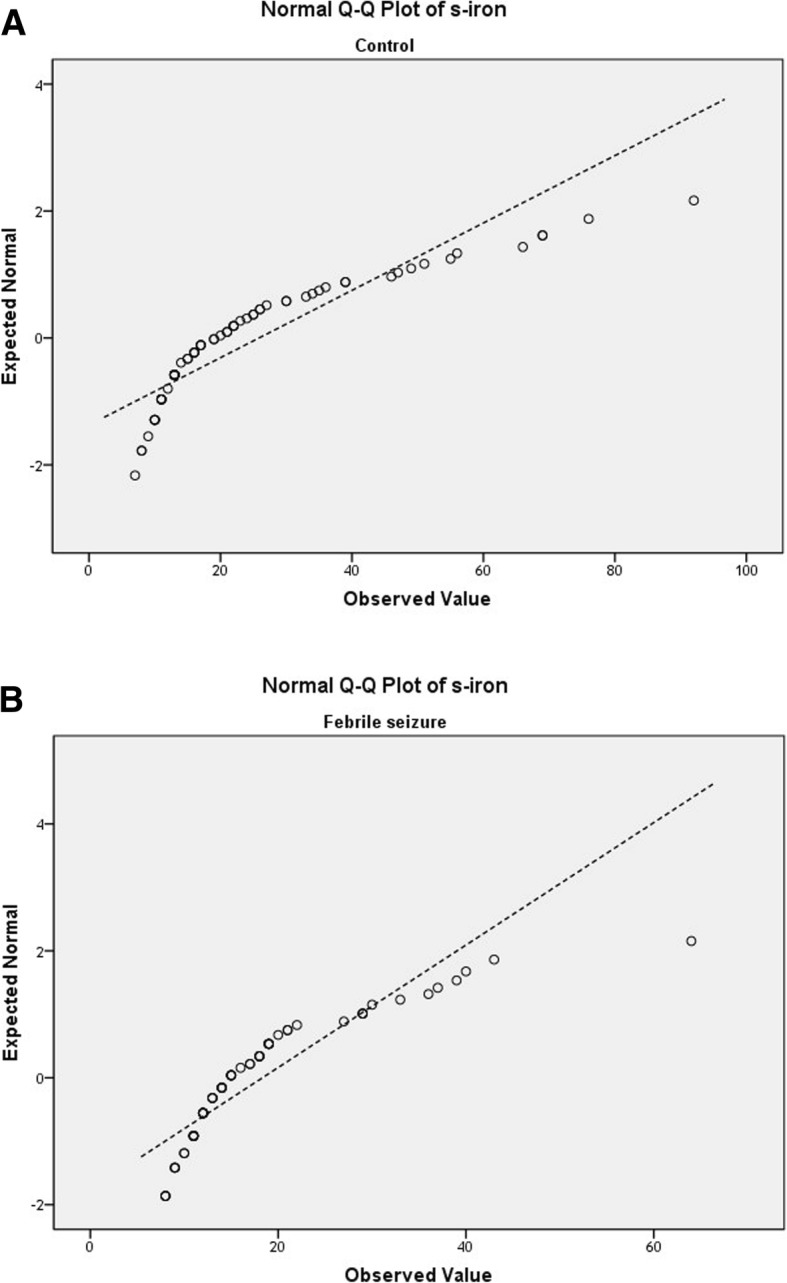
Q-Q plots of serum iron level in control group (**a**) and febrile seizure group (**b**) shows non-normal distribution

The collected data were analyzed using SPSS 21.0 statistical software. The Chi-square test was used for analysis of qualitative variables, while continuous variables were compared between case and control groups using independent-samples *t*-tests. After checking normality of the data by Shapiro-Wild test, we applied Mann-Whitney U tests for non-normal data and independent t tests for normally distributed data. The q-q- plots of hematocrit and serum iron level are presented in Figs. 1 and 2, representing normal and non-normal variables. Univariate analysis of all variables affecting febrile seizures were considered statistically significant with *P* < 0.10. Multiple logistic regression analysis was performed to examine the relationship between iron deficiency and development of febrile seizures. *P* < 0.05 was taken to indicate statistical significance.

## Results

The study population consisted of 63 children in the febrile seizure group and 65 children in the control group.). The mean age was 27.1 ± 13.5 months in febrile seizure group and 22.8 ± 13.3 months in control group (*P* = 0.07). The mean hemoglobin levels were 12.27 ± 0.75 g/dL in the febrile seizure group and 12.16 ± 1.01 g/dL in the control group; the difference was not significant. Comparison of the demographic and clinical characteristics between the two groups showed that body temperature was slightly higher in the febrile seizure group than the control group (Table 1). There were no differences in age, gender, or duration of fever between the two groups. The most common causes of febrile illness were upper respiratory infection in the febrile seizure group (61.9%) and pneumonia in the control group (43.1%).

**Table 1:** Demographic data and causes of fever in children with febrile seizures and control group

	Febrile seizures (*n* = 63)	Control (*n* = 65)	*P*-value
Age	27.1 ± 13.5	22.8 ± 13.3	0.07
Sex			0.22
Male	35 (55.6%)	29 (44.6%)	
Female	38 (44.4%)	36 (55.4%)	
Duration of fever	3.7 ± 1.9	4.0 ± 2.0	0.26
Peak body temperature	39.5 ± 0.8	39.1 ± 0.9	0.05
Causes of fever			
URI	46 (61.9%)	20 (15.4%)	
Pneumonia	8 (12.7%)	28 (43.1%)	
Viral illness	9 (14.3%)	1 (1.5%)	
Gastroenteritis	0	7 (10.8%)	
Lymphadenitis	0	3 (4.6%)	
UTI	0	6 (9.2%)	

*URI* Upper respiratory infection, *UTI* Urinary tract infection

Among children aged 6 to 24 months, two of 65 children in the control group and none of 63 children in the febrile seizure group had anemia. Two older children aged 24 to 60 months in each group had anemia; the difference was not statistically significant (9.1% vs. 8.7%, respectively, *P* > 0.05). Table 2 presents a summary of variable indices of iron status in the febrile seizure and control groups. Serum iron (18.32 ± 10.36 μg/dL vs. 25.85 ± 18.84 μg/dL, respectively, *P* = 0.03), plasma ferritin (35.98 ± 19.36 ng/mL vs. 56.81 ± 41.51 ng/mL, respectively, *P* < 0.001), and transferrin saturation (5.70 ± 3.30% vs. 8.45 ± 6.37%, respectively, *P* = 0.01) were significantly lower in the febrile seizure group compared to the controls.

**Table 2:** Comparison of major indices for iron state in children with febrile seizures and control group

	Febrile seizure (*n* = 63)		Control (*n* = 65)		*P*-value
	mean	SD	mean	SD	
Red blood cells (*10 ^9 /L)	4.64	0.31	4.63	0.36	0.54
Hemoglobin (g/dL)	12.27	0.75	12.16	1.01	0.77
Hematocrit (%)^a^	35.96	2.08	35.81	2.52	0.72
Mean cell volume (fl)	77.54	3.23	77.41	3.95	0.75
Mean cell hemoglobin (pg/cell)	26.63	1.79	26.45	2.31	0.95
Mean cell hemoglobin concentration (g/dL)	34.15	0.90	33.82	1.84	0.49
Red cell distribution width (%)	13.25	0.76	13.54	1.46	0.49
Reticulocyte (%)	1.71	0.50	1.63	0.83	0.09
White blood cells (*10^9 /L)	12,172.70	5162.00	11,226.31	4354.23	0.18
Platelets	282.97	89.34	328.43	136.65	0.04
Serum iron (μg/dL)	18.32	10.36	25.85	18.84	0.03
Total iron binding capacity (μg/dL)	326.94	43.67	312.63	48.19	0.05
Ferritin (ng/mL)	35.98	19.36	56.81	41.51	< 0.001
Transferrin saturation (%)	5.70	3.30	8.45	6.37	0.01

^a^ Analysis using unpaired two-sided t-test, other variables were analyzed using Mann-Whitney U tests

Ferritin < 30 ng/mL (49.2% vs. 16.9%, respectively, *P* < 0.001) and serum iron < 22 ng/dL (79.4% vs. 55.4%, respectively, *P* = 0.004) were more prevalent in the febrile seizure group compared with the control group. Comparing the hematological variables between simple (*n* = 47) and complex (*n* = 16) febrile seizures, there were no differences in hemoglobin, serum iron, TIBC, ferritin, or transferrin saturation (Table 3). In addition, the proportion of children with transferrin saturation < 16% was higher in the febrile seizure group than the control group (95.4% vs. 86.1%, respectively, *P* = 0.01) (Table 4). Univariate analysis found that serum iron, ferritin, and transferrin saturation was significantly associated with increased risk of febrile seizures with *P* < 0.10. In multivariate logistic regression analysis with these significant variables, low serum iron < 22 ng/dL (odds ratio 3.42, 95% confidence interval [CI] 1.31–8.9, *P* = 0.012) and low plasma ferritin < 30 ng/mL (odds ratio 6.18, 95% CI 2.32–16.42, *P* < 0.001) were shown to increase the risk of developing febrile seizures (Table 5).

**Table 3:** Comparison of major indices for iron state in children with with simple febrile seizures and complex febrile seizures

	Simple febrile seizures (*n* = 47)		Complex febrile seizures (*n* = 16)		*P*-value
	mean	SD	mean	SD	
Red blood cells (*10 ^9 /L)	4.66	0.31	4.59	0.31	0.46
Hemoglobin (g/dL)	12.35	0.72	12.03	0.79	0.17
Hematocrit (%)	36.13	2.08	35.48	2.07	0.28
Mean cell volume (fl)	77.61	3.36	77.34	2.91	0.77
Mean cell hemoglobin (pg/cell)	26.76	1.95	26.26	1.17	0.33
Mean cell hemoglobin concentration (g/dL)	34.22	0.84	33.94	1.07	0.29
Red cell distribution width (%)	13.23	0.81	13.31	0.60	0.72
Reticulocyte (%)	1.69	0.45	1.75	0.63	0.69
White blood cells (*10^9 /L)	12,429.36	5207.71	11,418.75	5114.36	0.50
Platelets	292.15	90.34	256.00	83.20	0.16
Serum iron (μg/dL)^a^	17.13	9.02	21.81	13.31	0.76
Total iron binding capacity (μg/dL)	324.94	46.46	332.81	34.83	0.54
Ferritin (ng/mL)	34.49	17.11	40.38	24.98	0.30
Transferrin saturation (%)^a^	5.41	3.04	6.57	3.95	0.67

^a^ Analysis using Mann-Whitney U test, other variables were analyzed using unpaired two-sided t-tests

**Table 4:** Proportion of major indices for iron state in children with febrile seizures and control group

	Febrile seizures (*n* = 63)	Control (*n* = 65)	*P*-value
Transferrin saturation < 16%	62 (95.4%)	56 (86.1%)	0.01
Ferritin < 30 ng/mL	31 (49.2%)	11 (16.9%)	< 0.001
Serum iron < 22 ng/mL	50 (79.4%)	36 (55.4%)	0.001

**Table 5:** Multivariate logistic regression analysis adjusted for sex and age

	Odd ratio	Lower 95% CI	Upper 95% CI	*P*-value
Serum iron < 22 μg/dL	3.42	1.31	8.90	0.01
Total iron binding capacity (μg/dL)	0.27	0.02	2.45	0.24
Ferritin < 30 ng/mL	6.18	2.32	16.42	< 0.01
Transferrin saturation < 16%	4.02	0.40	40.65	0.24

## Discussion

The results of this prospective case–control study indicated that iron deficiency, but not iron deficiency anemia, was more prevalent in children with febrile seizures compared to controls with febrile illness but no seizures. In multivariate logistic analysis, low serum iron and plasma ferritin were shown to be related to increased risk of febrile seizures.

Iron deficiency anemia occurs at similar ages to febrile seizures and is a prevalent problem, especially in developing countries, where 44–66% of children under the age of 4 years are anemic, with half of these cases attributed to iron deficiency anemia [26, 27].

The association between iron deficiency and febrile seizure has been examined in a number of studies, but the results remain controversial.

In a case–control study performed in 1996, Pisacane et al. [13] reported that iron deficiency anemia was significantly more common in febrile seizure cases (30%) than in hospital (14%) and population (12%) controls. Subsequent case–control studies from Iran and India also indicated an elevated prevalence of iron deficiency anemia in children with febrile seizures [13, 14, 28, 29]. Meanwhile, a Canadian study showed that children with febrile seizures were twice as likely to have iron deficiency (plasma ferritin level ≤ 30 ng/dL) as were those with febrile illness alone (OR, 1.84; 95% CI, 1.02–3.31), but there was no significant difference in proportion of anemia between the two groups [30]. Papageorgiou et al. [17] also reported that low plasma ferritin <30 ng/dL was more frequent in febrile seizure cases than in controls (24% vs. 4%, respectively, *P* = 0.004) in Greece. In a study performed in Jordan, mean ferritin level was significantly lower in cases with first febrile seizures than in controls [15]. In contrast, Amirsalari et al. [31] and Bidabadi et al. [20] reported no significant association between iron deficiency status and febrile seizures. Kobrinsky et al. [19] even suggested that iron deficiency may protect against the development of febrile seizures. Following these conflicting results, four recent meta-analyses indicated that iron deficiency anemia significantly increased the risk of febrile seizures with an OR of 1.27–3.78 [25–28]. In the meta-analysis by Kwak et al. [32], iron deficiency anemia, diagnosed based on plasma ferritin (OR 3.78; 95% CI, 1.80–7.94; *P* < 0.001) or MCV (OR 2.08; 95% CI, 1.36–3.17; *P* = 0.001), was associated with febrile seizures. Karimi et al. [33] performed subgroup analyses of simple febrile seizures and first febrile seizures, and obtained an OR of 2.98 (95% CI, 1.67–5.31) and 2.23 (95% CI, 1.33–3.73), respectively. Nasehi et al. [34] also reported an increased risk of febrile seizures in children with iron-deficiency anemia (OR = 1.27, 95% CI, 1.03–1.56), although the ferritin level did not differ significantly between the two groups in their meta-analysis. Another subgroup meta-analysis according to the prevalence of anemia found a greater risk of febrile seizures in areas with a low or moderate prevalence of iron deficiency anemia versus a high prevalence [35]. In the present study, iron deficiency, defined by a low ferritin level (< 30 ng/mL) or low serum iron (< 22 ng/dL), was associated with an increased risk of febrile seizures, although iron-deficiency anemia was not associated with febrile seizures. The discrepancies in the association of febrile seizures with anemia/iron deficiency state may be attributable to differences in ethnic background, socio-economic status, and accompanying nutritional status, as well as to the definitions of anemia and iron deficiency status used in the different studies.

Notably, the overall prevalence of iron deficiency anemia in the present study was 4.7% (6/128), which was much lower than in previous studies performed in the Middle East. The low prevalence of iron deficiency anemia may reflect improved nutritional status in children in South Korea. Most of the patients were from the capital, Seoul, which is a megacity with a high quality of life. The small number of patients with anemia may have led to a lack of association between iron deficiency anemia and febrile seizures. However, iron deficiency itself was found to increase the risk of febrile seizures in the present study.

Iron is an essential nutrient for proper growth and development in children. Iron deficiency interferes with the function of many organs, leading to anemia, abnormal growth and behavior, cognitive deficits, altered thermoregulation, impaired physical performance, and immune dysfunction [12, 36]. The effects of iron deficiency on the developing brain have been identified in a variety of animal studies. Iron is important for catecholamine metabolism and for the various enzymes and neurotransmitters present in the central nervous system. Iron deficiency increases extracellular dopamine and norepinephrine levels in the caudate–putamen and decreases the levels of dopamine D1 and D2 receptors and monoamine transmitters [37]. Furthermore, iron deficiency in early life alters metabolism and neurotransmission in major brain structures, such as the basal ganglia and hippocampus, and disrupts myelination [38]. Infants aged 6–24 months with iron deficiency anemia are at risk for poorer cognitive, motor, socioemotional, and neurodevelopmental outcomes [39]. In addition, iron deficiency is associated with several neurological disorders, such as restless leg syndrome, breath-holding spells, and attention deficit hyperactivity disorders, which are associated with increased brain excitability. Recently, Rudy et al. [40] demonstrated that mice exposed postnatally to iron deficiency had a decreased seizure threshold and increased seizure susceptibility to certain types of seizures. The precise mechanism underlying the association between iron deficiency and brain hyperexcitability has not been fully elucidated, but the above evidence suggests that disruption of normal neurotransmitter activity and brain metabolism may predispose children with iron deficiency to increased risk of developing febrile seizures.

Iron status may also reflect general health, including nutrition, growth, and immunity in children. Poorer general health status may be associated with febrile seizures via low seizure threshold or frequent infection [41, 42].

The main limitation of the present study was the potential confounding effect of ferritin as an acute-phase reactant agent, which can interfere with identifying the influence of iron status on febrile seizures. However, patients in both the febrile seizure group and the control group were enrolled at the time of febrile illness, so it is supposed that the difference in ferritin level between the two groups would be significant.

The results of this study were noteworthy based on its prospective design and its location in East Asia, in contrast to most previous studies regarding this issue. It is suggested that iron deficiency is a significant issue for children worldwide, that clinicians should pay attention to.

## Conclusions

Iron deficiency, defined by low ferritin level or low serum iron, was associated with increased risk of febrile seizures. Therefore, in children with febrile seizures, we suggest that clinicians should be concerned for iron status even at normal hemoglobin levels. Further studies are required to determine the detailed pathomechanism underlying the association between iron deficiency and a lower seizure threshold. In addition, further prospective studies are needed to determine whether iron supplementation can prevent the occurrence of febrile seizures.

## Data Availability

The datasets generated and analyzed during the current study are not publicly available but may be available from the corresponding author on reasonable request.

## Abbreviations

CBC: Complete blood count
Hb: Hemoglobin
TIBC: Total iron binding capacity
URI: Upper respiratory infection
UTI: Urinary tract infection

## References

[1] Berg AT (1992). Febrile seizures and epilepsy: the contributions of epidemiology. Paediatr Perinat Epidemiol.

[2] Subcommittee on Febrile seizures, American Academy of Pediatrics (2011). Neurodiagnostic evaluation of the child with a simple febrile seizure. Pediatrics.

[3] Barlow WE, Davis RL, Glasser JW, Rhodes PH, Thompson RS, Mullooly JP (2001). The risk of seizures after receipt of whole-cell pertussis or measles, mumps, and rubella vaccine. N Engl J Med.

[4] Ganesh R, Janakiraman L (2008). Serum zinc levels in children with simple febrile seizure. Clin Pediatr (Phila).

[5] Graves RC, Oehler K, Tingle LE (2012). Febrile seizures: risks, evaluation, and prognosis. Am Fam Physician.

[6] Huang CC, Wang ST, Chang YC, Huang MC, Chi YC, Tsai JJ (1999). Risk factors for a first febrile convulsion in children: a population study in southern Taiwan. Epilepsia.

[7] Laina I, Syriopoulou VP, Daikos GL, Roma ES, Papageorgiou F, Kakourou T (2010). Febrile seizures and primary human herpesvirus 6 infection. Pediatr Neurol.

[8] Vestergaard M, Hviid A, Madsen KM, Wohlfahrt J, Thorsen P, Schendel D (2004). MMR vaccination and febrile seizures: evaluation of susceptible subgroups and long-term prognosis. JAMA.

[9] Byeon JH, Kim GH, Eun BL (2018). Prevalence, incidence, and recurrence of febrile Seizures in Korean children based on national registry data. J Clin Neurol.

[10] Han YJ, Chang GT (2007). Recent advances in febrile seizures. J Pediatr Korean Med.

[11] Rouault TA, Cooperman S (2006). Brain iron metabolism. Semin Pediatr Neurol.

[12] Beard JL (2001). Iron biology in immune function, muscle metabolism and neuronal functioning. J Nutr.

[13] Pisacane A, Sansone R, Impagliazzo N, Coppola A, Rolando P, D'apuzzo A (1996). Iron deficiency anaemia and febrile convulsions: case-control study in children under 2 years. Br Med J.

[14] Ghasemi F, Valizadeh F, Taee N (2014). Iron-deficiency anemia in children with febrile seizure: a case-control study. Iran J Child Neurol.

[15] Daoud AS, Batieha A, Abu-Ekteish F, Gharaibeh N, Ajlouni S, Hijazi S (2002). Iron status: a possible risk factor for the first febrile seizure. Epilepsia.

[16] Zareifar S, Hosseinzadeh HR, Cohan N (2012). Association between iron status and febrile seizures in children. Seizure.

[17] Papageorgiou V, Vargiami E, Kontopoulos E, Kardaras P, Economou M, Athanassiou-Mataxa M (2015). Association between iron deficiency and febrile seizures. Eur J Paediatr Neurol.

[18] Koksal AO, Ozdemir O, Buyukkaragoz B, Karaomerlioglu M, Bulus AD (2016). The association between plasma ferritin level and simple febrile seizures in children. J Pediatr Hematol Oncol.

[19] Kobrinsky NL, Yager JY, Cheang MS, Yatscoff RW, Tenenbein M (1995). Does iron deficiency raise the seizure threshold?. J Child Neurol.

[20] Bidabadi E, Mashouf M (2009). Association between iron deficiency anemia and first febrile convulsion: a case–control study. Seizure.

[21] Yousefichaijan P, Eghbali A, Rafeie M, Sharafkhah M, Zolfi M, Firouzifar M (2014). The relationship between iron deficiency anemia and simple febrile convulsion in children. J Pediatr Neurosci.

[22] Underwood BA (1985). Weaning practices in deprived environments: the weaning dilemma. Pediatrics.

[23] Berg AT, Shinnar S (1996). Complex febrile seizures. Epilepsia.

[24] Oski FA, Brugnara C, Nathan DG. A diagnostic approach to the anemic patients. In: Nathan DG, Orkin SH, editors. Nathan and Oski’s hematology of infancy and childhood. 5th ed. Philadelphia: W.B. Saunders Company; 1998.

[25] Phiri K, Calis J, Siyasiya A, Bates I, Brabin B, Van Hensbroek M (2009). New cut-off values for ferritin and soluble transferrin receptor for the assessment of iron deficiency in children in a high infection pressure area. J Clin Path.

[26] DeMaeyer E, Adiels-Tegman M (1985). The prevalence of anaemia in the world. World Health Stat Q.

[27] Florentino R, Guirriec RM, Stekel A (1984). Prevalence of nutritional anemia in infancy and childhood with emphasis on developing countries. Nutrition in infancy and childhood.

[28] Sherjil A, us Saeed Z, Shehzad S, Amjad R (2010). Iron deficiency anaemia--a risk factor for febrile seizures in children. J Ayub Med Coll Abbottabad.

[29] Srinivasa S, Reddy SP (2014). Iron deficiency anemia in children with simple febrile seizures-a cohort study. Curr Pediatr Res.

[30] Hartfield DS, Tan J, Yager JY, Rosychuk RJ, Spady D, Haines C (2009). The association between iron deficiency and febrile seizures in childhood. Clin Pediatr (Phila).

[31] Amirsalari S, Doust ZTK, Ahmadi M, Sabouri A, Kavemanesh Z, Afsharpeyman S (2010). Relationship between iron deficiency anemia and febrile seizures. Iran J Child Neurol.

[32] Kwak BO, Kim K, Kim SN, Lee R (2017). Relationship between iron deficiency anemia and febrile seizures in children: a systematic review and meta-analysis. Seizure.

[33] Karimi P, Badfar G, Soleymani A, Khorshidi A (2018). Association of iron deficiency anemia and febrile seizure in Asia: a systematic review and meta-analysis. Iran J Neonatoly.

[34] Nasehi MM, Abbaskhanian A, Salehi Omran MR (2013). Association between iron deficiency anemia and febrile seizure: a systematic review and meta-analysis. J Pediatr Rev.

[35] Habibian N, Alipour A, Rezaianzadeh A (2014). Association between iron deficiency anemia and febrile convulsion in 3-to 60-month-old children: a systematic review and meta-analysis. Iran J Med Sci.

[36] Beard J (2003). Iron deficiency alters brain development and functioning, 2. J Nutr.

[37] Beard JL, Chen Q, Connor J, Jones BC (1994). Altered monamine metabolism in caudate-putamen of iron-deficient rats. Pharmacol Biochem Behav.

[38] Lozoff B, Georgieff MK (2006). Iron deficiency and brain development. Semin Pediatr Neurol.

[39] Lozoff B, Beard J, Connor J, Felt B, Georgieff M, Schallert T (2006). Long-lasting neural and behavioral effects of iron deficiency in infancy. Nutr Rev.

[40] Rudy M, Mayer-Proschel M (2017). Iron deficiency affects seizure susceptibility in a time-and sex-specific manner. ASN Neuro.

[41] Hackett R, Iype T (2001). Malnutrition and childhood epilepsy in developing countries. Seizure.

[42] Rantala H, Uhari M, Tuokko H (1990). Viral infections and recurrences of febrile convulsions. J Pediatr.

